# Mesoporous carbon nitride supported MgO for enhanced CO_2_ capture

**DOI:** 10.1007/s11356-023-26013-5

**Published:** 2023-03-03

**Authors:** Zakaria Refaat, Mohamed El Saied, Ahmed O. Abo El Naga, Seham A. Shaban, Hanaa B. Hassan, Mohamed Refaat Shehata, Fathy Y. El Kady

**Affiliations:** 1 Catalysis Department, Refining Division, Egyptian Petroleum Research Institute, Nasr City, 11727 Cairo Egypt; 2 Chemistry Department, Faculty of Science, Cairo University, Giza, Egypt

**Keywords:** Mesoporous carbon nitride, CO_2_ capture, Global warming, MgO

## Abstract

The growing concern about the environmental consequences of anthropogenic CO_2_ emissions significantly stimulated the research of low-cost, efficient, and recyclable solid adsorbents for CO_2_ capture. In this work, a series of MgO-supported mesoporous carbon nitride adsorbents with different MgO contents (xMgO/MCN) was prepared using a facile process. The obtained materials were tested for CO_2_ capture from 10 vol% CO_2_ mixture gas with N_2_ using a fixed bed adsorber at atmospheric pressure. At 25 ºC, the bare MCN support and unsupported MgO samples demonstrated CO_2_ capture capacities of 0.99, and 0.74 mmol g^−1^, respectively, which were lower than those of the xMgO/MCN composites.The incorporation of MgO into the MCN improved the CO_2_ uptake, and the 20MgO/MCN exhibited the highest CO_2_ capture capacity of 1.15 mmol g^−1^ at 25 °C. The improved performance of the 20MgO/MCN nanohybrid can be possibly assigned to the presence of high content of highly dispersed MgO NPs along with its improved textural properties in terms of high specific surface area (215 m^2^g^−1^), large pore volume (0.22 cm^3^g^−1^), and abundant mesoporous structure. The efffects of temperature and CO_2_ flow rate were also investigated on the CO_2_ capture performance of 20MgO/MCN. Temperature was found to have a negative influence on the CO_2_ capture capacity of the 20MgO/MCN, which decreased from 1.15 to 0.65 mmol g^−1^with temperature rise from 25 C to 150º C, due to the endothermicity of the process. Similarly, the capture capacity decreased from 1.15 to 0.54 mmol g^−1^ with the increase of the flow rate from 50 to 200 ml minute^−1^ respectively. Importantly, 20MgO/MCN showed excellent reusability with consistent CO_2_ capture capacity over five sequential sorption–desorption cycles, suggesting its suitability for the practical capture of CO_2_.

## Introduction

Anthropogenic carbon dioxide exhausts are believed to be the principal cause behind global warming, which is one of the most serious existential threats to human survival (Pang et al. 2021; Ekanayake et al. 2021; Creamer et al. 2018; Li et al. 2013). The concentration of CO_2_ in the atmosphere has experienced a remarkable increase from the pre-industrial level of 280 or less ppm to above 400 ppm in the present day (Yamori and Ghannoum 2022). It is estimated that carbon dioxide alone is responsible for roughly two-thirds of global warming (Ekanayake et al. 2021; Creamer et al. 2018; Li et al. 2013). Most CO_2_ emissions come predominantly from the burning of fossil fuels in power stations for electric power generation and vehicles for transportation purposes (Ekanayake et al. 2021; Lakhi et al. 2015a; Pu et al. 2021). Unfortunately, there will be no real change in the world's energy mix in the future (Xiao et al. 2021). The world will remain primarily dependent on fossil fuels to fulfill its energy needs for many decades to come*.* This, and the expected growth of the world's demand for energy in the future, renders the instantaneous cessation of anthropogenic CO_2_ emissions unattainable. Therefore, ecologists and environmental scientists are striving hard to develop new and efficient strategies for CO_2_ capture from the global atmosphere and enhancing the efficiency of fossil fuel burning (Ekanayake et al. 2021; Li et al. 2013; Pu et al. 2021).

Today, absorption using liquid amines (mainly monoethanolamine) is the prevailing commercial technique for CO_2_ capture as a consequence of its high CO_2_ capacity and fast reaction rate (Ekanayake et al. 2021; Li et al. 2013; Pu et al. 2021). Unfortunately, liquid amines are highly toxic and highly volatile, besides being corrosive to equipment parts (Lakhi et al. 2015b; Pu et al. 2021). Importantly, the regeneration of the amine solution for long-term recyclability requires a tedious, costly, and energy-consuming procedure, constituting the hardest challenge impeding the continued development and use of this approach (Ekanayake et al. 2021; Li et al. 2013; Pu et al. 2021). In this context, the adsorption using porous solid materials has earned considerable attention as a green and economic alternative to alleviating the difficulties related to using liquid amines absorption technology for carbon dioxide capture (Pu et al. 2021; Hu et al. 2022). Compared to liquid amines, solid adsorbents offer several prominent advantages, such as ease of handling, low operational expenditure, non-corrosively, more practical working conditions*,* no liquid waste disposal, and more importantly, facile and cost-effective regeneration procedure (Pang et al. 2021; Li et al. 2013; Lakhi et al. 2015a; Ha et al. 2021; Wang et al. 2022; Vaghasia et al. 2022; Lai et al. 2022). High CO_2_ adsorption capacity, high selectivity, rapid adsorption kinetics, facile and scalable synthesis method, and superb recycling performance are indispensable features for a porous material to be considered as a promising candidate for CO_2_ capture. So far, several porous solids have been evaluated regarding their efficiency as adsorbents for the selective capture of CO_2_. Examples include metal–organic frameworks (Vaghasia et al. 2022; Lin et al. 2022), hydrotalcite (Faria et al. 2022), zeolites (Shen et al. 2022; Gonzalez-Olmos et al. 2022), metal oxides (Papalas et al. 2021; Gao et al. 2021; Mutch et al. 2018; Long et al. 2022; Bahadoran et al. 2022a) and porous carbon (Singh et al. 2019; Zhang et al. 2019).

Magnesium oxide (MgO) stands out as one of the most auspicious contender materials for CO_2_ capture thanks to its effective CO_2_ capture over an expansive temperature spectrum together with reduced energy demands for CO_2_ regeneration (Pang et al. 2021; Li et al. 2013; Pu et al. 2021; Guo et al. 2022). Besides, MgO is environmentally benign and can be prepared in bulk amounts at a low cost due to its abundant occurrence in nature (Pang et al. 2021; Bahadoran et al. 2022b).

However, MgO suffers from a challenging shortcoming: the formation of a carbonate layer on its surface, which may limit the accessibility of the CO_2_ molecules to the unoccupied active adsorption sites, leading to poor CO_2_ capture performance (Papalas et al. 2021; Jo et al. 2017). However, most of the MgO-based sorbents formerly documented in the literature suffer from moderate CO_2_ capture performance and slow sorption kinetics (Cui et al. 2020). It was reported that the unsatisfactory CO_2_ capture efficiency and sorption kinetics of MgO-based sorbents are ascribed to their limited specific surface area along with the formation of a carbonate layer on its surface, which may limit the accessibility of the CO_2_ molecules to the unoccupied active adsorption sites (Papalas et al. 2021; Jo et al. 2017, Li and Zeng 2017). Moreover, the regeneration of the MgO-based adsorbents usually took place at relatively high temperatures (400–500 °C). Such high-temperature treatment will inevitably lead to the sintering and aggregation of the MgO nanoparticles and drastic loss of the sorbent surface area, impeding their recyclability in cycling tests (Li and Zeng 2017). Therefore, to develop high-performance MgO-based sorbents for carbon capture all of these issues must be addressed. A conceivable way to improve the CO_2_ capture performance and stability of MgO might be to incorporate them into suitable high surface area robust support materials (Papalas et al. 2021; Gao et al. 2017a). Compositing with highly porous support can uniformly disperse MgO nanoparticles, leading to more exposed binding active sites, thus improving the capture efficiency (Li and Zeng 2017). More importantly, the metal–support interaction can fix the MgO species tightly, averting their aggregation and enhancing the adsorbent stability (Li and Zeng 2017). Recently, several adsorbents based on MgO loaded onto various supporting materials, such as porous carbon (Li et al. 2013; Pu et al. 2021), alumina (Li et al. 2010a), TiO_2_ (Jeon et al. 2012), zeolites (Signorile et al. 2019), and mesoporous silica (Wei et al. 2004) have been broadly studied and utilized as adsorbents to efficiently capture CO_2_ from gas streams.

Carbon nitride is a type of two-dimensional material that is being researched more and more due to its interesting combination of incomparable properties, such as easy and inexpensive synthesis, environmental benignity**,** inherent basicity, low density, biocompatibility, hardness, and superior electronic and electric features (Idris and Devaraj 2019; Oh et al. 2015; Liu et al. 2021; Park et al. 2017; Ha et al. 2019). In addition, the existence of nitrogen atoms in the structural framework of carbon nitride can not only endow the material with heightened thermal, chemical, and mechanical stabilities but also provide its surface with inherent basic functionalities (Jin et al. 2009; Ekanayake et al. 2021; Guo et al. 2022; Jo et al. 2017). The combination of these attractive attributes has endowed carbon nitride with a high potential of being employed in miscellaneous important applications, such as energy storage and conversion, catalysis, sensing, and gas capture. Unfortunately, the traditional techniques employed for the production of carbon nitride yielded a highly condensed material with very low porosity and small surface area, which is of limited usage in many applications, such as CO_2_ capture, that require materials with elevated porosity (Idris and Devaraj 2019; Nazari et al. 2021; Ha et al. 2019). Attending this situation, considerable endeavors have been paid to develop alternative synthetic strategies to fabricate carbon nitride materials with highly porous structures. The introduction of porosity along with surface area augmentation would ensure the exposure of a great number of highly reachable active sites, thus paving the way for the use of porous carbon nitrides in a wide range of practical applications, which are beyond the capability of condensed analogs (Jo et al. 2017; Jeon et al. 2012). It has been shown that highly porous carbon nitrides can act serve as high-performance adsorbents for CO_2_ capture (Deng et al. 2012; Park et al. 2017; Lakhi et al. 2015a).

In the current work, novel adsorbent materials for CO_2_ capture have been synthesized by compositing MgO nanoparticles with mesoporous g-C_3_N_4_ support (MCN). To the best of our knowledge, the hybridization of MCN and MgO has not been evaluated for CO_2_ adsorption before. For this purpose, mesoporous carbon nitride material (MCN) was first synthesized by the hard template approach, using SBA-15 material as the sacrificial template, ethylenediamine (EDA), and carbon tetrachloride (CTC) as the sources of nitrogen, and carbon, respectively. The obtained MCN was further utilized as a support material for developing a series of xMgO/MCN adsorbents with different MgO loadings (5–25 wt%) by a modified ultrasonic-assisted method. The high BET surface area, abundant mesoporous structure, and a large number of highly accessible active sites conferred MgO/MCN nanohybrids with high CO_2_ capture capacity and good cycling performance.

## Experimental

### Materials and methods

Triblock copolymer Pluronic P123 ((PEO)_20_(PPO)_70_(PEO)_20_), tetraethyl orthosilicate (TEOS, Si(OC_2_H_5_)_4_, 98%), ethylenediamine (EDA, C_2_H_4_ (NH_2_)_2_, ≥ 99%), hydrofluoric acid (HF, 40% in water), and magnesium nitrate hexahydrate (Mg(NO_3_)_2_.6 H_2_O, 99%) were purchased from Sigma-Aldrich. Hexadecyltrimethyl-ammonium bromide (CTAB, CH_3_(CH_2_)_15_N(Br)(CH_3_)_3_, 99%), and hydrochloric acid (HCl, 36.5%) were supplied from MERCK. Absolute ethanol (C_2_H_5_OH, 99%), sodium hydroxide (NaOH ≥ 99.0%),and 1000 PPM Magnesium standard solution for Atomic Absorption spectrometer were obtained from Scharlau. Carbon tetrachloride (CTC, CCl_4_, ≥ 99.5%) was purchased from Pubchem. The materials were used as received without additional purification. The certified 10/90 vol% CO_2_ /N_2_ pre-mixed gas cylinder was purchased from ETICO gas.

### Synthesis of mesoporous SBA-15

The mesoporous silica SBA-15 was synthesized following the synthetic route described formerly by Yang and co-workers (Liu et al. 2009).

### Synthesis of mesoporous carbon nitride

Mesoporous carbon nitride (MCN) was prepared by the polymerization of CTC and EDA using SBA-15 as a hard template (Deng et al. 2012). In a typical synthesis, a mixture of 1.35 g of EDA and 3.0 g of CTC was prepared, and 0.5 g of SBA-15 was added to it and refluxed at 90 °C while stirring for 6 h. The obtained dark-brown colored solid was transferred to the oven for drying at 80 °C for 12 h after being separated via centrifugation. The dried product was calcined in a nitrogen flow at 800 °C for 5 h and eventually soaked into 10 wt% HF aqueous solution under magnetic stirring for 24 h at room temperature to remove the remained template and obtain the targeted MCN material.

### Synthesis of magnesium oxide

The basic magnesium oxide was synthesized by precipitation reaction between magnesium nitrate as a starting material and sodium hydroxide as a precipitating agent following the recipe described by Shin and co-workers (Wahab et al. 2007) with amendments. In this procedure: 50 ml of 1.0 M Mg (NO_3_)_2_.6H_2_O solution and 50 ml of 2.0 M sodium hydroxide solution were prepared separately. The sodium hydroxide solution was added dropwise to the magnesium nitrate solution under vigorous stirring. After a few minutes of stirring, a dense white precipitate of magnesium hydroxide is formed. The synthesis mixture was heated to 80 °C and stirred continuously for 2 h, before being left to stand under quiescent conditions at ambient temperature for 12 h. The white precipitate was harvested by centrifugation and, in turn, washed copiously with purified water till neutrality, and vacuum dried at 60 °C for 4 h. Ultimately, MgO NPs were obtained, after calcining the dried precursor in the air at 550 °C for 4 h (5 °C min^−1^).

### Synthesis of MgO/MCN nanohybrids

Finally, a series of MgO NPs embedded inside the MCN nanocomposites with different MgO loadings (5–25 wt.%) was prepared as follows: 1.0 g of as-prepared MCN was dispersed in 50 ml of absolute ethanol by alternate sonication and stirring for 3.0 h with 0.5 h for each step. Then, a specific amount of MgO was added slowly to the MCN dispersion with sonication. After being stirred at ambient conditions for 12 h, the mixture was evaporated to dryness on a rotary evaporator at 80 °C for 4 h. The resulting solids were dried at 105 °C for 12 h and eventually calcined at 500 °C in N_2_ flow for 2 h. The obtained solid adsorbents were as designated xMgO/MCN, where x corresponds to the weight percentage of MgO.

### Characterization

Patterns of X-ray diffraction (XRD) were analysed under ambient conditions by Cu Kα radiation at 40 kV and 150 mA with a Bruker AXS D8 diffractometer. The high-angle XRD measurements were done at a scan speed of 4 º per minute from 10˚ to 80˚ while the low-angle scan speed was 0.045˚ per second from 0.5º to 8.

For surface area, pore diameter and pore volume,The samples were first degassed at 250˚C for more than 5 h, then the measurements were done at the temperature of liquid nitrogen (77 K) using a Quanta Chrome NOVA 3200 S sorption analyzer. The procedure of BET was used for the calculation of the specific surface area (S_BET_) for each sample, while the BJH method was used for the determination of the pore size distributions of the samples from the desorption branch.

The pretreatment of the samples for (TEM) is essential. In this process, the samples were dispersed in ethanol and sonicated for 5 min, and then dried and deposited on grids made of Cu coated with films of holey carbon. For the measurements, a JEOL JEM 2100F microscope was used for the analysis at 200 kV.

Fourier-transform infrared spectroscopy (FT-IR) data were obtained by 32 scans within the frequency range of 400–4000 cm^−1^ and 4 cm^−1^ resolution using a ATI unicam (Mattson 936) Bench Top Spectrometer after dilution of the samples by physical mixing between the powdered samples and KBr_(S)_ while Sentera spectrometer was used for Raman Spectroscopy measurements.

Elemental analysis was performed by Perkin Elmer Atomic Absorption spectrometer (PinAAcle 500) using Lumina Magnesium Hollow cathode lamp (operate 6, Max 15). Thermo Scientific X-ray photoelectron spectroscopy (XPS) with a monochrome Al Kα source as an X-ray source was used to investigate the surface elemental composition and bondinig characteristic of the catalyst. The Belcat II apparatus was used to measure CO_2_-TPD. Prior to analysis, the sample was cleaned by heating it at 550 °C for 4 h. After cooling, the sample was saturated with CO_2_ and heated at a rate of 5 °C /min under helium flow. A thermal conductivity detector was used to determine the amount of desorbed CO_2_ gas (TCD).

### Adsorption Experiment

**Fig. 1:**
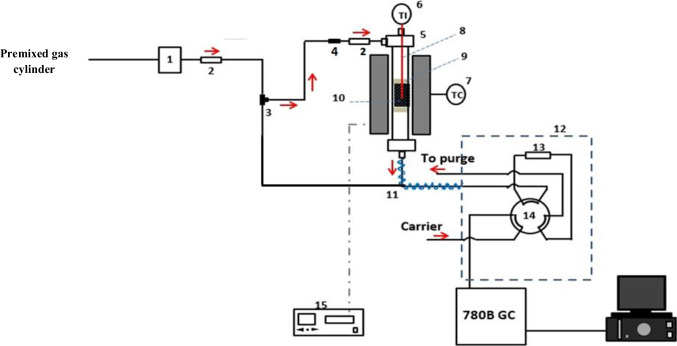
A schematic diagram for the fixed bed flow reactor. 1: Mass flow controller; 2: non-return valve; 3: Three-way valve, 4: filter; 5: stainless steel reactor; 6: temperature indicator; 7: temperature controller; 8: thermocouple; 9: quartz wool; 10: catalyst bed; 11: heating tap; 12: hot box; 13; sample loop; 14: six-port valve; 15: furnace controller

The CO_2_ dynamic adsorption experiments were carried out in a homemade continuous flow fixed bed reactor at atmospheric pressure. A schematic illustration of this unit is depicted in Fig. 1. the reactor is constructed from a 316 stainless steel tube having 30 cm total length and 10 mm inner diameter. The reactor was heated to the required operating temperature by employing a vertical split-tube furnace. During capture tests, the adsorbent bed temperature was constantly recorded by a K-type thermocouple inserted in a thermowell mounted in the middle of the reactor inside the adsorbent bed. Mass flow controllers were used to regulate the flow rates of the incoming gases; meanwhile, the flow rate of the reactor effluent was recorded by a mass flow meter. In a typical experiment, 0.5 g of the adsorbent (particle size = 200–400 μm) was charged in the middle of the reactor, corresponding to a total packing length of around 9 mm. In the remaining vacant region above and below the adsorbent bed, inert glass beads were placed to ensure homogenous distribution of the incoming gases and to save the adsorbent particles from escaping the bed. After being packed inside the reactor, the adsorbent was pre-dried in a pure nitrogen flow (100 ml min^−1^) at 200 ˚C and atmospheric pressure for 2 h. After the reactor was adjusted to the desired operating temperature, CO_2_/N_2_ gas mixture at 50 ml min^−1^ was introduced from a certified premixed gas cylinder of 10 vol% CO_2_ and 90 vol% N_2_. The effluent stream of the reactor outlet was analyzed immediately by an online Agilent 780-B gas chromatograph provided with a thermal conductivity detector (TCD). The outlet CO_2_ concentration was monitored by an online Agilent 780-B gas chromatograph equipped with a thermal conductivity detector (TCD) (Kangwanwatana et al. 2013). Please note that each capture test was repeated thrice**,** and the mean values were considered.

## Results and discussion

### Characterization

**Fig. 2:**
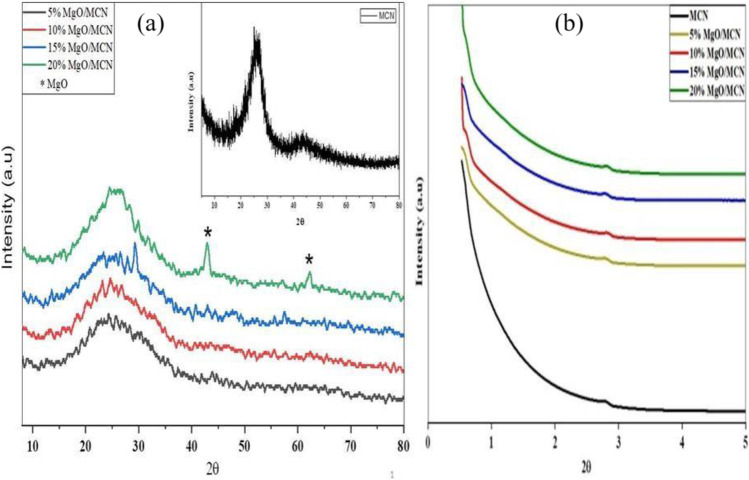
(**a**) High angle XRD, and (**b**) low angle XRD of MCN and xMgO/MCN

As shown in Table 1, the actual MgO loadings, as determined by AAS, were in satisfactory accord with the theoretical ones. Figure 2 a. depicts wide-angle XRD patterns of MCN, and xMgO/MCN composites with different MgO contents. The XRD patterns of the pristine MCN material, and the xMgO/MCN nanohybrids, manifested only a broad reflection positioned at a 2θ value of approximately 25.3° that can be well indexed to the (1 0 0) crystallographic plane of graphite (JCPDS 87–1526) (Liu et al. 2016). No diffraction lines corresponding to crystalline magnesium-containing species were seen in composites with MgO contents up to 20 wt%, signifying that magnesium species were uniformly distributed in the MCN matrix (Fathi et al. 2020). As can be noticed, when the MgO content reached 20 wt%, the (0 0 2) and (2 0 2) reflections of MgO emerged at 2θ of 44° and 62°, respectively (JCPDS No. 75–1525). On the other hand at low angles (Fig. 2 b), mesoporous carbon nitride and a series of MgO-supported MCN demonstrated a strong peak at 2θ = 0.5, which can be indexed to the (1 0 0) reflection line, and weak signals at 2θ = 2.0 corresponding to (2 0 0) reflections, denoting the formation of well-ordered hexagonal mesoporous material with space group p6mm. This result is evidence that mesoporous carbon nitride with an ordered 2D hexagonal symmetric structure was successfully synthesized using the SBA-15 template, and the structure of MCN remained unchanged with the introduction of MgO nanoparticles.

**Table 1:** Surface characteristics of the produced materials

Materials	Surface area (m^2^ g^−1^)	Pore volume (cc g^−1^)	MgO content (wt%)
MCN	729.2	1.03	–
5MgO / MCN	419.7	0.60	4.67
10MgO / MCN	352.3	0.39	10.24
15MgO / MCN	322.2	0.36	14.84
20MgO / MCN	215.0	0.22	19.62

**Fig. 3:**
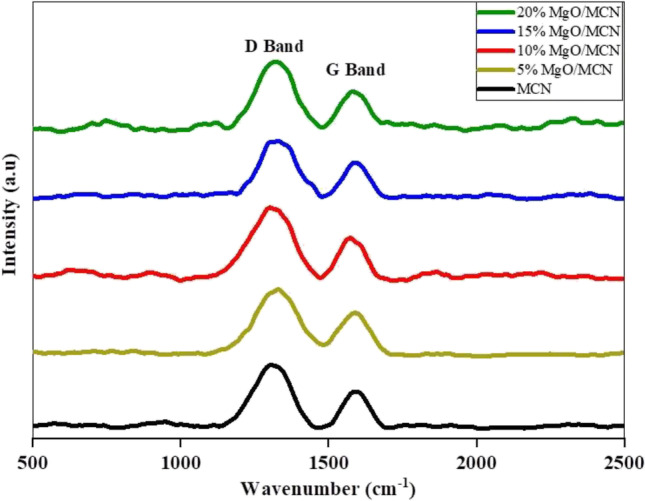
Raman spectroscopy of MCN and xMgO/MCN

Raman spectroscopy was used to scrutinize the graphitic and disorder characters of the synthesized samples (Mao et al. 2022; Abo El Naga et al. 2019). Raman spectra of the MCN and xMgO/MCN are depicted in Fig. 3. The resultant spectra of the bare MCN demonstrated two signals located at approximately 1350 and 1580 cm^−1^, which were designated to the disordered (D) and graphitic (G) modes of graphite analogous materials (Li et al. 2010b; Abo El Naga et al. 2018, Youssef et al. 2020, Saied et al. 2022). The Raman spectra of xMgO/MCN composites were similar to that of MCN**.**

**Fig. 4:**
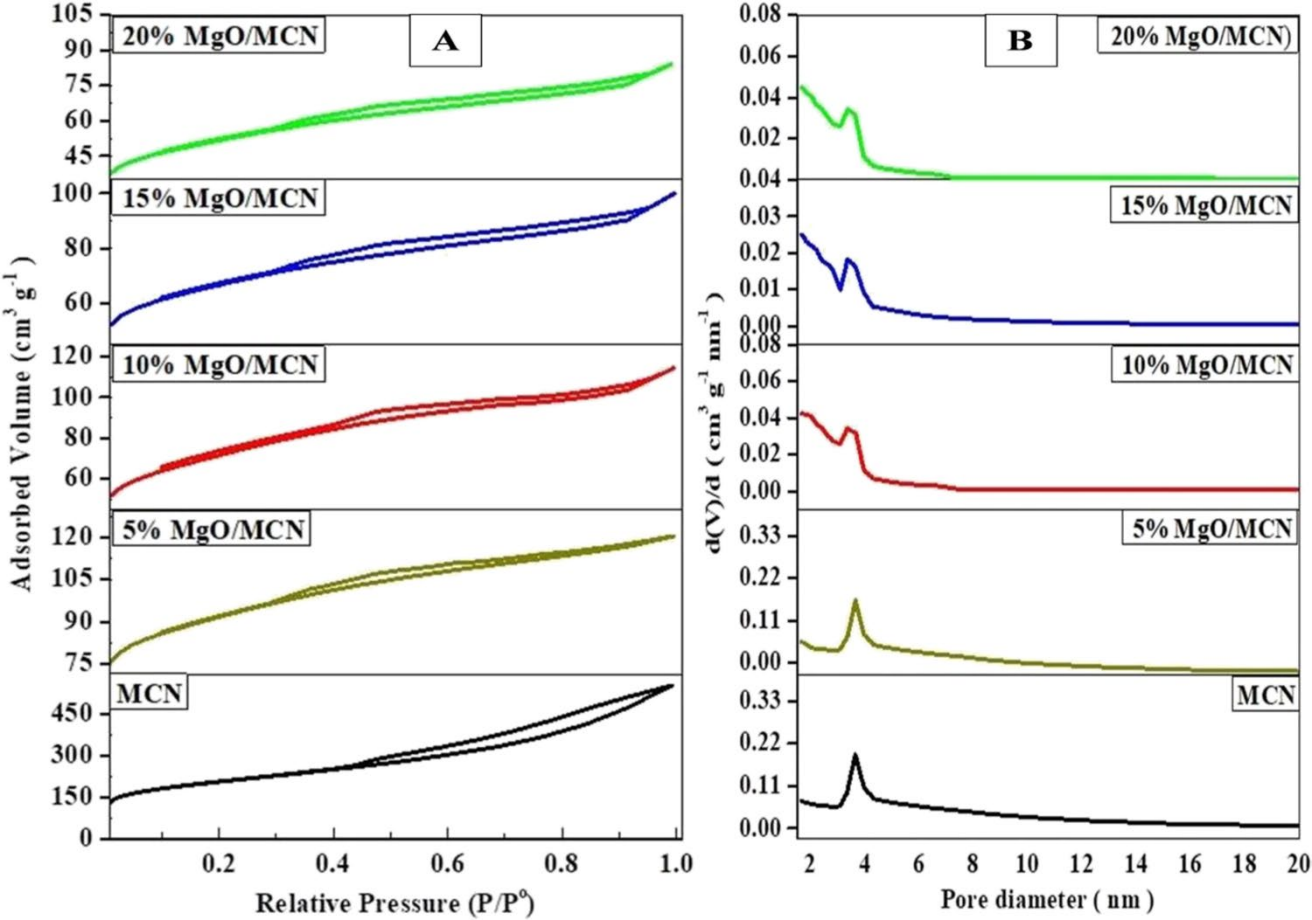
(**A**) N_2_ adsorption–desorption isotherms and (**B**) pore size distributions of MCN and xMgO/MCN

The nitrogen-adsorption desorption isotherms and the corresponding pore size distribution curves of MCN, and xMgO/MCN composites are given in Fig. 4 (a-b) As is depicted in this figure, the nitrogen adsorption–desorption isotherm of MCN is of type IV, verifying the mesoporous nature of the material. MCN reveals an H_2_ hysteresis loop with the capillary condensation at relative pressures (p/p˚) ranging from 0.40 to 0.9. No substantial differences were observed in the shape of the N_2_ adsorption isotherm of the pristine support upon the introduction of MgO nanoparticles, see Fig. 4 (a-b), verifying that the original mesoporous structure of MCN material was well preserved throughout the adsorbent preparation steps. The results agreed well with the information in the XRD analysis. A progressive decline in the amount of the adsorbed N_2_ is observed with the increase in MgO loading due to the partial occupation of the pores by MgO nanoparticles. As can be noticed in Fig. 4 (a-b), the pore size distribution of the MCN and xMgO/MCN adsorbents also confirmed their mesoporous structures. The textural parameters of the synthesized materials, including BET-specific surface area, total pore volume, are outlined in Table 1. From Table 1, it can be noted that MCN has a BET surface area and total pore volume of about 729.2 m^2^ g^−1^ and 1.03 cm^3^ g^−1^, respectively. Nevertheless, raising the MgO loading level resulted in a progressive drop in the BET surface area and pore volume of the MgO loaded materials, which can be ascribed to the partial occupation of the porous structure of MCN by MgO nanoparticles.

**Fig. 5:**
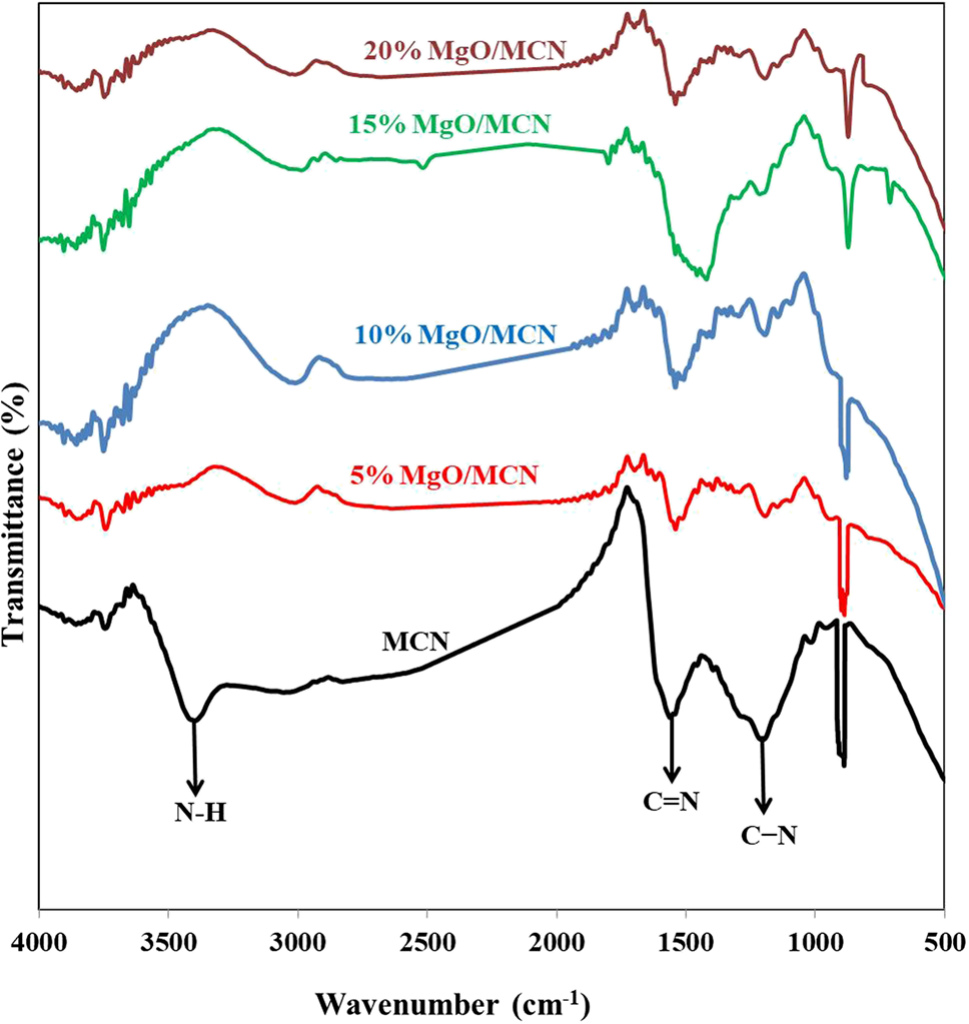
FTIR of MCN and xMgO/MCN

To gain more structural information regarding the synthesized MCN-based materials, FT-IR spectra were recorded. The resulting spectra of MCN and xMg/MCN are demonstrated in Fig. 5. The spectra of MCN show bands at 3386.53 cm^−1^, 1534.16 cm^−1^, and 1177.38 cm^−1^, which are the result of the stretching modes of N–H bonds in –NH_2_ or = NH groups, C = N bonds, and aromatic C − N bonds, respectively (Dodangeh et al. 2021; Fathi et al. 2020). Furthermore, the stretching absorption peak of the − C≡N groups in the vicinity of 2200 cm^−1^ was not observed, implying that MCN contains s-triazine building units in their skeleton. Interestingly, xMgO/MCN composites yielded a similar spectrum to the parent MCN. This revealed that the chemical structure of the MCN was preserved during the loading of MgO NPs (Pham et al. 2022; Zheng et al. 2020). Notably, the intensities of the peaks at 1534.16 cm^−1^ and 1177.88 cm^−1^ were weakened obviously after MgO loading, which is presumably a consequence of the coordination bonding between the Mg and N atom in MCN (Madona and Sridevi 2022; Ge et al. 2018). The peak at 887.13 cm^−1^ was related to the bending form of vibration which results from heptazine rings, indicating that the prepared MCN contains heptazine units (Deng and Li 2018).

**Fig. 6:**
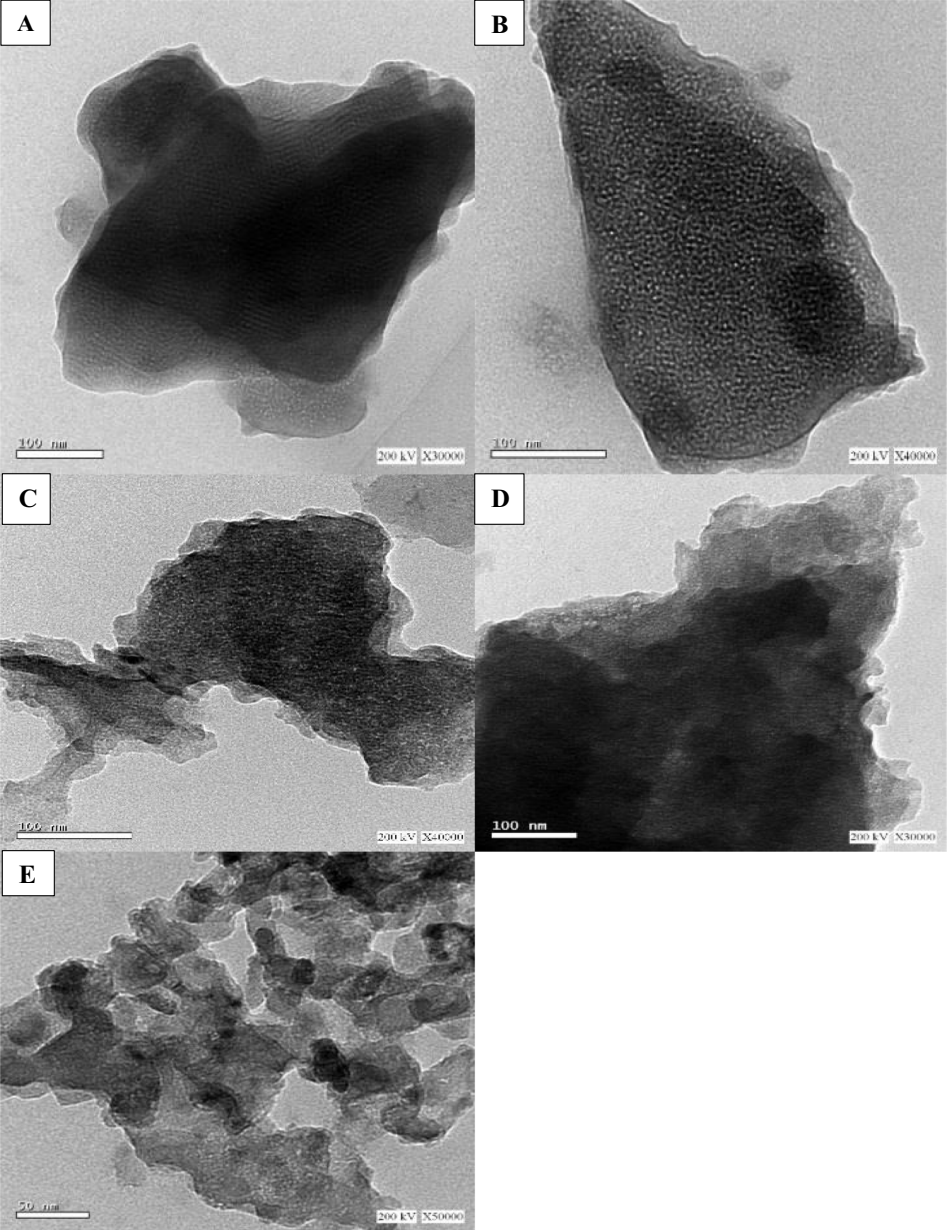
HRTEM of (**a**) MCN, (**b**) 5MgO/MCN, (**c**) 10MgO/MCN, (**d**) 15MgO/MCN, and (**e**) 20MgO/MCN

The morphological features of the prepared mesoporous carbon nitride and xMgO/MCN are depicted in Fig. 6 (a–e). According to displayed TEM images, an open structure and a crystallin nature for the pure carbon nitride can be noticed. Additionally, all xMgO/MCN samples displayed uniformly and well-dispersed particles of MgO in the mesoporous carbon nitride skeletons. All structures have also revealed porous nature however, a higher level of porosity can be seen in the pristine carbon nitride sample. These observations coincide with the BET results mentioned above. A progressive decline in the BET surface area and pore volume of the MgO loaded materials was observed with increasing the MgO loading level as a consequence of the partial occupation of the porous structure of MCN by MgO nanoparticles.

**Fig. 7:**
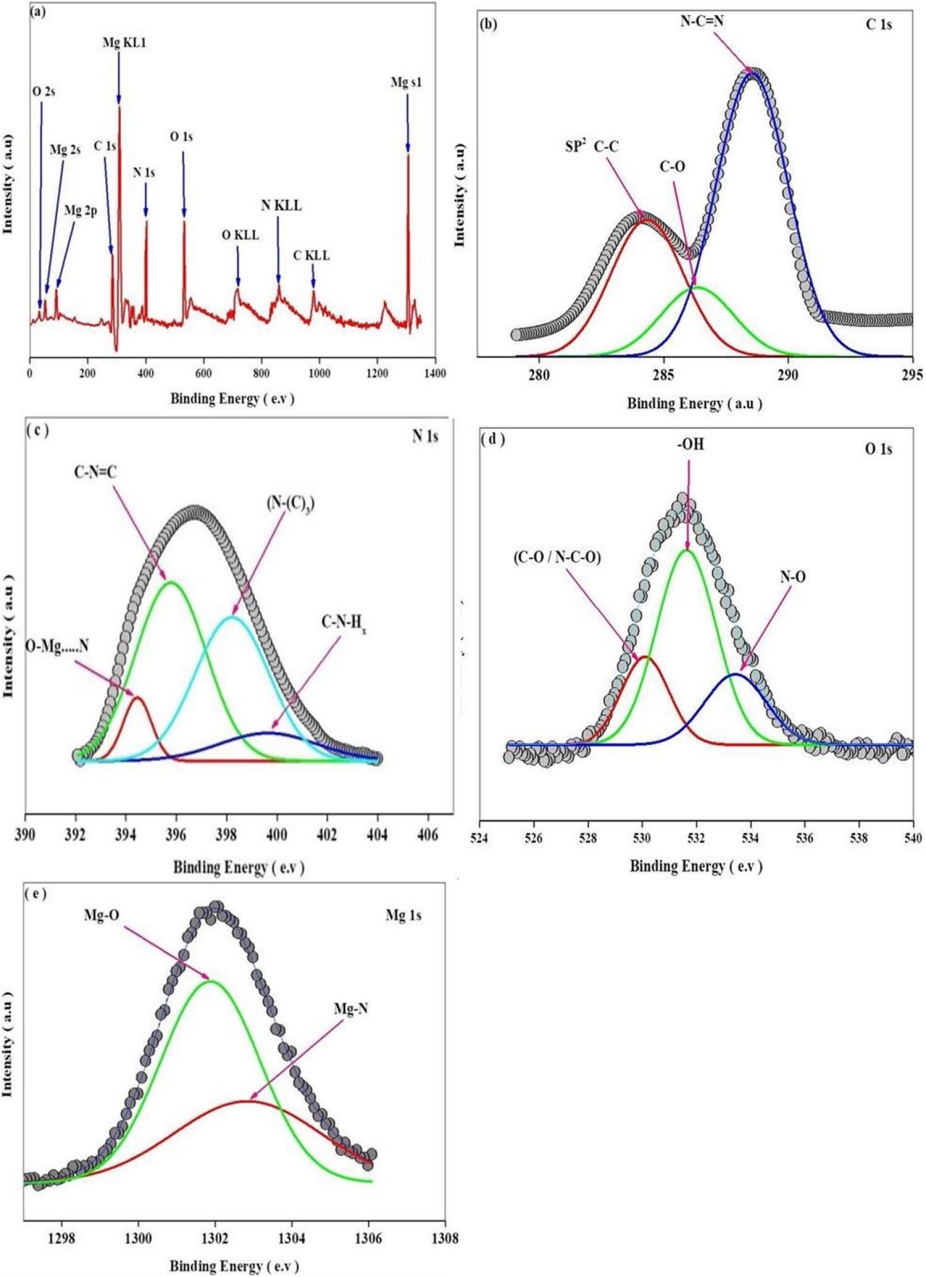
XPS spectra of (**a**) survey, (**b**) C1s, (**c**) N1s, (**d**) O1s, and (**e**) Mg1s for 20MgO/MCN

The X-ray photoelectron spectroscopy (XPS) analysis was conducted to evaluate the surface elemental composition and bonding characteristics of 20MgO/MCN (Mohamed et al. 2020). Figure 7 shows the spectra of (a) the survey, (b) C1s, (c) N1s, (d) O1s, and (e) Mg1s for 20MgO/MCN. The low-resolution survey spectrum (Fig. 7 a) demonstrated the presence of C, O, N, and Mg in the sample, which were the main constituent atoms of the 20 MgO/MCN nanocomposite. The signals at binding energies of 51 and 89 eV could be related to the Mg 2 s and Mg 2p peaks, respectively, which are characteristic of MgO (Kapilashrami et al. 2010). Additionally, the signal at 300 eV related to Mg KL1 (Liu et al. 2022a), and the Mg 1 s was responsible for the peak centered at 1303 eV (Singh and Rath 2015). The peaks at 43 and 533 eV correspond to O2s and O 1 s, respectively (Ge et al. 2018; Schulze et al. 2004)و while the peak at 733.6 eV is related to O KLL (Vinu et al. 2005). Carbon peaks are visible at 284.8 and 980 eV as C1s (Mao and Jiang 2019b) and C KLL (Vinu et al. 2005), respectively. The peaks at 398 and 850 eV correspond to N 1 s (Mao and Jiang 2019b) and N KLL (Vinu et al. 2005), respectively. As illustrated in Fig. 7 b, the high-resolution C1s spectrum can be divided into three peaks at 284.4, 285.8, and 288.3 eV, respectively, correspond to SP^2^ hybridized C–C bonds in the MCN skeleton, C-O bonds between MCN and MgO, and the binding of SP^2^ hybridized carbon with nitrogen in the heterocycle (N–C = N) of aromatic rings in the MCN skeleton (Yamada et al. 2014; Deng and Li 2018). Figure 7 c displays four peaks in the high-resolution N 1 s spectrum. The peaks centering at 394.8, 395.9, 398.8, and 399.5 eV refer to nitrogen bonded to magnesium (Mg-N bond), nitrogen bonded to carbon that is bi-coordinated (C = N–C), tri-coordinated (N-(C)_3_), and nitrogen of amine groups (C-N-H_x_,x = 1, 2), respectively (Ge et al. 2018). The high-resolution O1s depicted in Fig. 7 d show three peaks related to the oxygen bound to carbon (O-C-N), surface adsorbed oxygen (OH groups), and oxygen bounded to nitrogen (O-N–C) centered at 530, 531.9, and 533.6 eV, respectively (Fu et al. 2017; Lei et al. 2022; Liu et al. 2022b). In addition, Mg 1 s high-resolution spectra showed two peaks characteristic of Mg-O at 1301.7 eV and Mg-N bond between MCN and MgO at 1302.5 eV, as illustrated in Fig. 7 e (Mao and Jiang 2019a). Finally, we can conclude from XPS analysis that the only elements present on the surface of the catalyst are Mg, O, N, and C. The MgO interacts with the MCN surface via Mg-N, N–O, and C-O coordination bonds, which agree with the XRD and FTIR analysis.

**Fig. 8:**
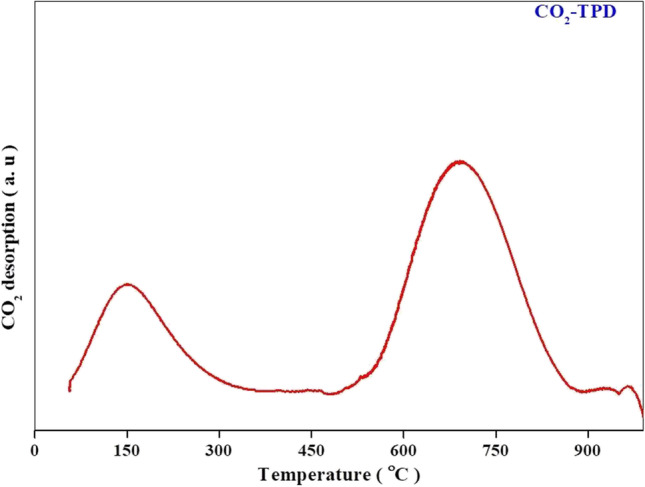
CO_2_-TPD analysis of 20% MgO/MCN

The CO_2_-TPD analysis of the 20MgO/MCN catalyst was carried out to investigate its basicity and base strength, as well as to gain insight into the basic sites distributed in the catalyst.The CO_2_-TPD analysis pattern of the catalyst at various temperatures was measured and illustrated in Fig. 8. The sample's total CO_2_ desorption was 9.672 mmol/g, which corresponds to the basic strength of the catalyst. The pattern has two distinct peaks that correspond to weak and strong basic active sites, respectively. The desorbed peak of weak active sites at 150 ºC is related to nitrogen species in the MCN structure that physisorb CO_2_. Furthermore, the desorption peak occurs at 700 °C due to CO_2_ chemisorption on the MgO basic sites (Deng and Li 2018).

### CO_2_ capture

#### Effect of MgO loading

**Fig. 9:**
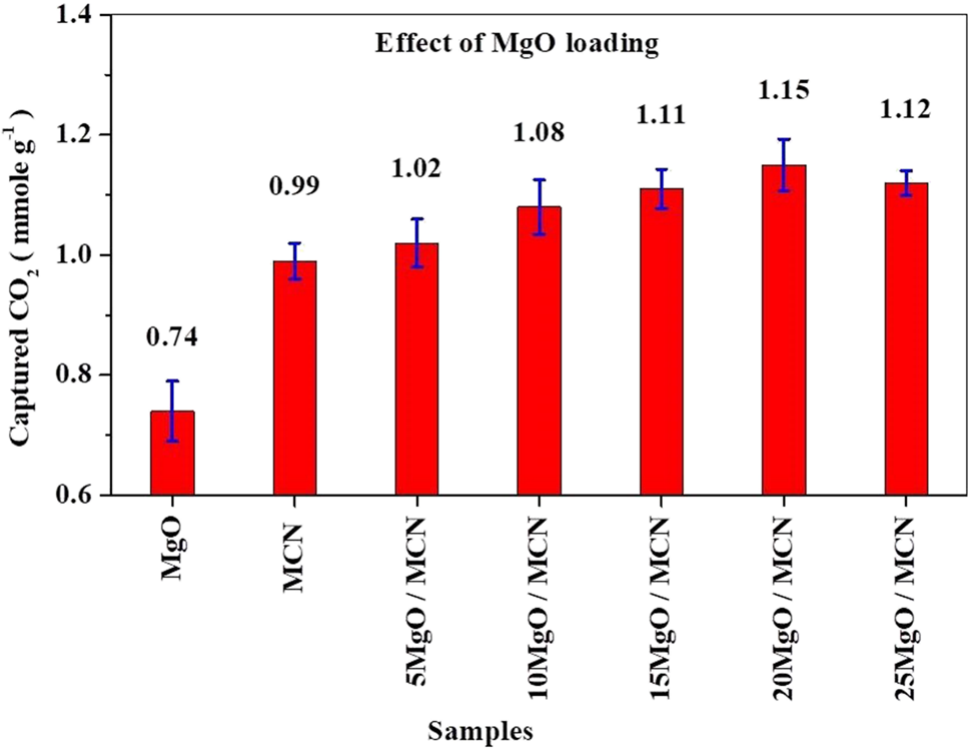
CO_2_ adsorption capacities of MCN, MgO and xMgO/MCN samples

To elucidate the effect of MgO content on CO_2_ capture performance of the supported MgO adsorbents, the xMgO/MCN adsorbents with different MgO contents are tested in 10% CO_2_ and 90% N_2_ at 25 °C. Figure 9 represents the CO_2_ capture, in mmol of CO_2_ per gram of xMgO/MCN, for each examined sample. It is evident from the figure that the incorporation of MgO into the MCN improved the CO_2_ uptake. Under the same conditions, the CO_2_ capture amount increased with increasing the MgO from 0.99 mmol g^−1^ for the bare support to 1.15 mmol g^−1^ for 20MgO/MCN. However, further content to 25 wt. % caused an obvious decline in the CO_2_ capture amount. The incorporation of MgO can efficiently improve the basic properties of the porous support material and offer a higher number of effective basic adsorption sites for binding acidic CO_2_ molecules (Pu et al. 2021). Accordingly, all xMgO/MCN adsorbents have higher affinities toward CO_2_ as compared to the pristine MCN. The decrease of adsorption efficiency with increasing MgO content above 20 wt.% was probabely caused by the blockage of the pores of MCN with excessive MgO (Pu et al. 2021; Nowrouzi et al. 2018). These findings are consistent with those of other MgO-supported adsorbents reported in the literature (Pu et al. 2021). Furthermore, it is worth mentioning that the CO_2_ capture capacity of the bulk MgO sample (0.74 mmol g^−1^) was far inferior to that of supported MgO adsorbents, highlighting the indispensable role that porous structure has on the CO_2_ uptake performance. From all of the previous observations, the optimium catalyst ratio for this process is 20MgO/MCN. Following, 20MgO/MCN was selected to evaluate the effect of adsorption conditions on the removal of CO_2_ from the simulated air.

#### Effect of adsorption temperature

**Fig. 10:**
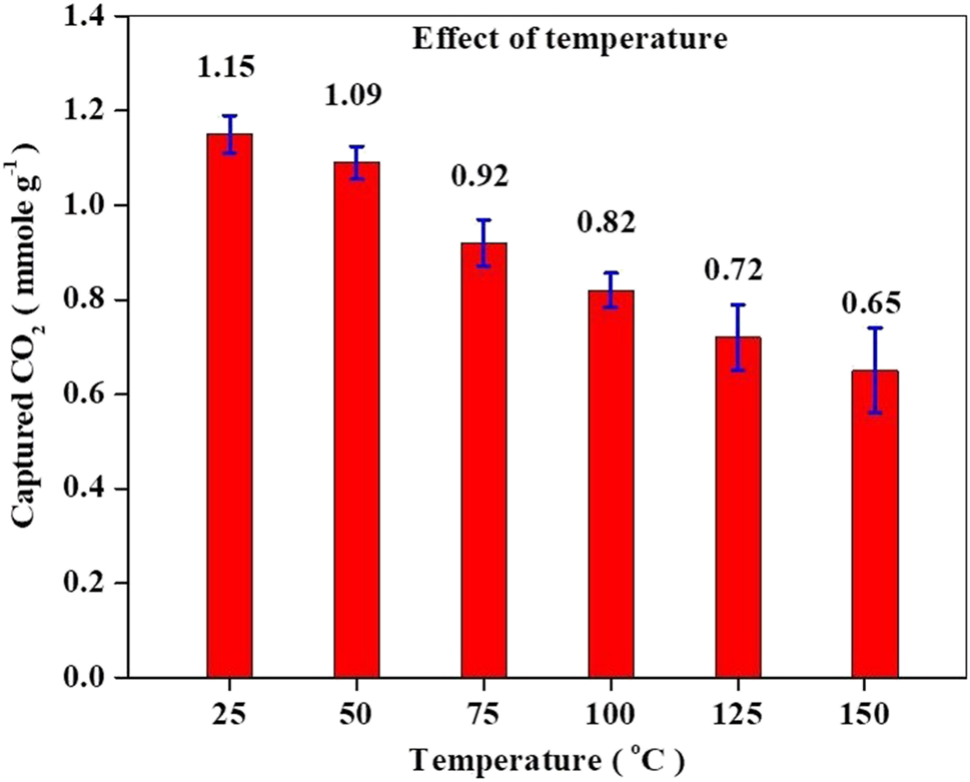
CO_2_ adsorption capacity of 20MgO/MCN at different temperatures

The effect of CO_2_ sorption temperatures on the capture performance of 20MgO/MCN was studied by varying the temperature between 25 and 150 ºC, and the experimental results are presented in Fig. 10. It was observed that the highest CO_2_ capture amount was obtained at 25 ºC. This means that high temperature is not beneficial for CO_2_ adsorption over 20 Mg/MCN, which is likely due to the endothermicity of the adsorption process (Pu et al. 2021; Li et al. 2013). A similar trend for the change in CO_2_ uptake capacity with temperature has been reported before in literature (Pu et al. 2021; Li et al. 2013; García et al. 2008; Osler et al. 2017). However, it is worth noting that 20MgO/MCN composite still maintains acceptable CO_2_ adsorption capacity at 150 °C, revealing the effectiveness of composite for working over a wide operating temperature range.

#### Effect of CO_2_ flow rate

**Fig. 11:**
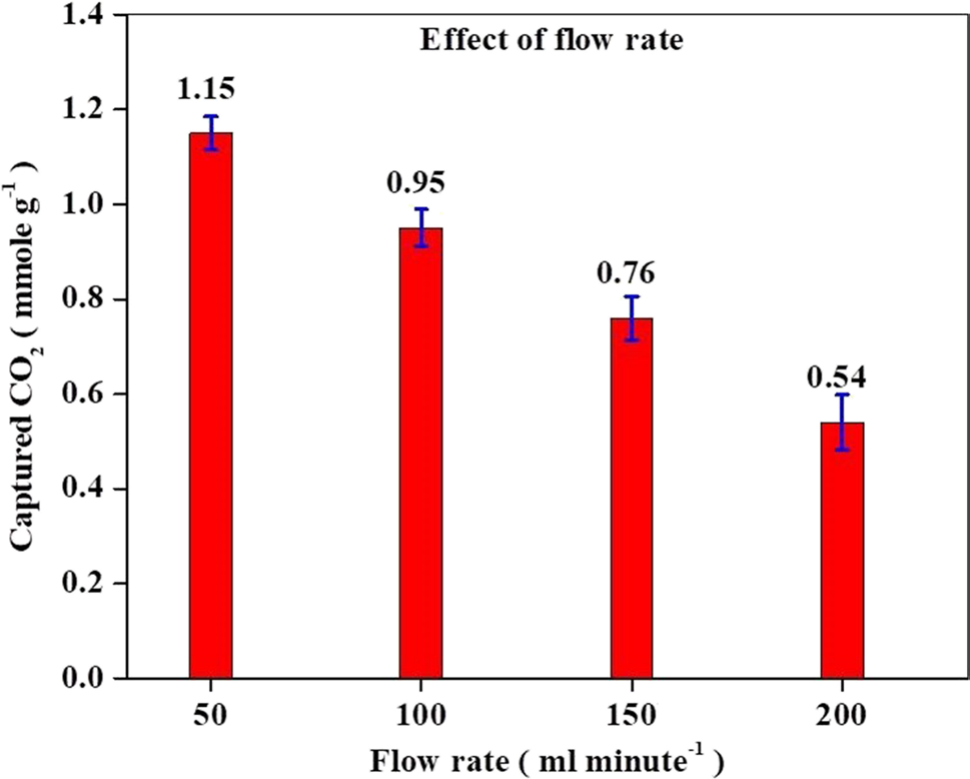
CO_2_ adsorption capacity of 20MgO/MCN at different CO_2_ flow rates

The CO_2_ capture performance of the 20MgO/MCN composite was also evaluated at different gas flow rates of CO_2_. (50–200 ml min^−1^) at 25 °C. The experimental results in Fig. 11 showed that the efficiency of CO_2_ removal was adversely affected by the increase in CO_2_ flow rate; the CO_2_ adsorption capacity of the 20MgO/MCN dropped from 1.15 to 0.54 mmole g^−1^ with the increment of the CO_2_ flow rate from 50 to 200 ml min^−1^. This decrease might be because, with an increasing CO_2_ flow rate, the residence time between CO_2_ and the adsorbent decreases, resulting in lower CO_2_ capture efficiency (Osler et al. 2017).

#### Cyclic adsorption performance

**Fig. 12:**
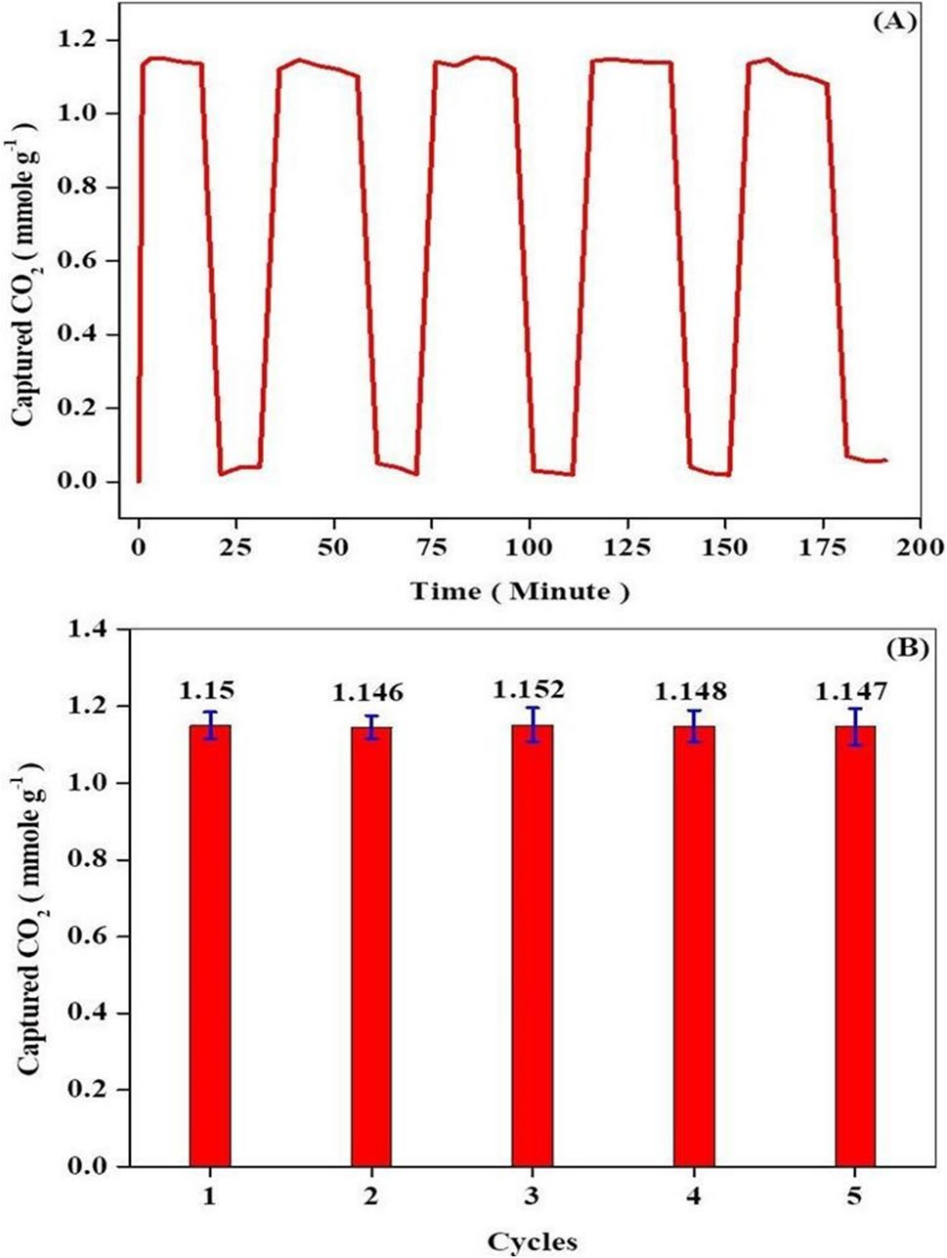
(**a**) CO_2_ adsorption capacity of 20% MgO/MCN at Cyclic adsorption performance, (**b**) cycles stability

From a practical application standpoint*,* cyclic performance is another significant concern that must be taken into consideration when evaluating the adsorption performance of potential CO_2_ sorbents. Herein, the adsorption behavior of 20MgO/MCN was appraised for five sequential experiments of CO_2_ sorption–desorption. CO_2_ adsorption experiments were conducted at 25 °C with 10 vol % CO_2_/ 90 vol % N_2_ at 50 ml min^−1^; meanwhile, the desorption experiments were conducted with 100 ml/min of N_2_ at 400 °C. The results of these experiments are shown in Fig. 12 a. It can be seen that almost all the adsorbed CO_2_ molecules were released during the desorption process, indicating that the CO_2_ adsorption on 20MgO/MCN is fully reversible. In addition, it was also observed from Fig. 12 b. that after five sequential sorption–desorption cycles, the CO_2_ capture capacity of 20MgO/MCN kept almost constant, parading the excellent operational stability and recyclability of MgO/MCN during CO_2_ capture for practical applications.

**Fig. 13:**
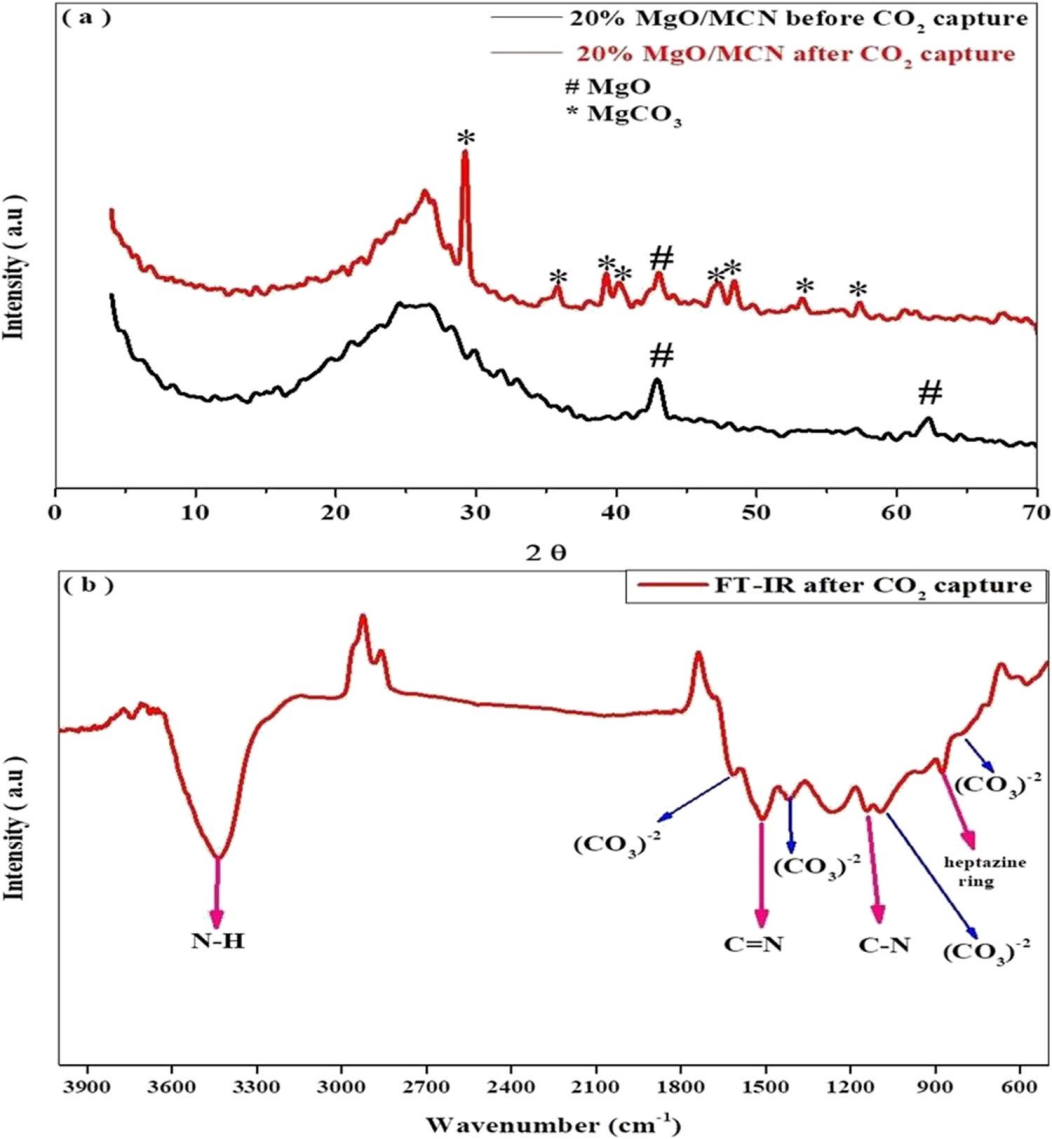
(**a**) XRD of 20MgO/MCN before and after CO_2_ capture, (**b**) FT-IR of 20MgO/MCN after CO_2_ capture

Following a cyclic stability study, XRD and FT-IR were performed to evaluate its stability and elucidate the mechanism of CO_2_ capture. As shown in Fig. 13 a, the pattern after CO_2_ capture preserved the characteristic peak of MCN at 2θ = 25° and the peak of MgO at 44°. In addition, new diffraction lines were observed, which could be ascribed to MgCO_3_.3H_2_O (JCPDS Card 020–0669) (Bhagiyalakshmi et al. 2010; Chen et al. 2020). The formation of magnesium carbonate was a result of the reaction between MgO and CO_2_ (Eq. 1), indicating that bulk chemical phase conversion from MgO to MgCO_3_ took place during CO_2_ capture. In other words, CO_2_ uptake occurred via bulk chemical transformation rather than surface adsorption.

1 $$\mathrm{MgO}+{\mathrm{CO}}_{2}\to \mathrm{Mg}-\mathrm{O}\dots \dots {\mathrm{CO}}_{2(\mathrm{ads})}$$

Figure 13 b depicts the FT-IR analysis of the sample after CO_2_ capture. The IR spectrogram revealed the preservation of the peaks that correspond to the catalyst's skeleton, such as the peaks at 3395, 1535, and 1179 cm^−1^, which corresponded to N–H bonds in -NH_2_ or = NH groups, C = N bonds, and aromatic C-N bonds, respectively (Dodangeh et al. 2021; Fathi et al. 2020). Furthermore, the peak at 888 cm^-1^ is related to heptazine ring bending (Deng and Li 2018). After CO_2_ capture, new peaks were found as a result of the carbonate group. These characteristic peaks centered at 850, 1120, and 1480 cm^−1^, which are assigned to the bending vibrations, symmetric stretching vibration, and asymmetric stretching vibrations of the carbonate group, respectively (Botha and Strydom 2003). Moreover, the peak at 1649 cm^−1^ corresponded to the asymmetric stretch of bidentate carbonates (Gao et al. 2017b).

#### Comparison study

The CO_2_ capture performance of 20MgO/MCN was compared with those of other MgO-based CO_2_ adsorbents reported recently in the literature (Table 2). In this study, a CO_2_ capture amount of 1.15 mmol g^−1^ was achieved using 20Mg/MCN, which is on par with or even exceeding those of other counterpart adsorbents listed in Table 2. Based on the aforementioned results, such improved performance of 20MgO/MCN can be possibly assigned to the presence of high content of highly dispersed MgO NPs along with its improved textural properties in terms of high specific surface area (215 m^2^ g^−1^), large pore volume (0.22 cm^3^ g^−1^), and mesoporous nature. Thus, 20MgO/MCN exhibited the benefits of good CO_2_ capture amount, wide operating temperature range, and excellent reusability, suggesting its suitability for the practical capture of CO_2_.

**Table 2:** Comparison of CO_2_ adsorption capacity of some MgO-based materials

Samples	Calcination temperature (°C)	CO_2_ adsorption temperature (°C)	Adsorption capacity(mmol g^−1^)	Adsorption pressure (atm)	Ref
MgO/Al _2_O_3_	**600**	**25**	**0.73**	**1**	(Kim et al. 2014)
Calcinated magnesite	**500**	**60**	**1.82**	**4**	(Yang et al. 2018)
MgO	**400**	**50**	**0.81**	**1**	(Song et al. 2015)
MgO/k- SBA	**540**	**20**	**0.91**	**1**	(Zukal et al. 2013)
MG–480–42–13.8	**480**	**60**	**0.77**	**1**	(Sun et al. 2016)
MgO(solvothermal)	**550**	**90**	**0.36**	**1**	(Zhao et al. 2011)
MgO/Al-SBA	**450**	**25**	**1.36**	**1**	(Zhao et al. 2012)
MgO	**400**	**50**	**1.59**	**1**	(Song et al. 2016)
MgO + Cs_2_CO_3_	**-**	**300**	**1.9**	**0.5**	(Liu et al. 2013)
MgO-Al_2_O_3_ aerogel	**600**	**200**	**0.5**	**1**	(Han et al. 2014)
MgO /LiNO_3_	**450**	**325**	**1.17**	**1**	(Vu et al. 2014)
MgO /LiNO_3_	**450**	**300**	**1.45**	**1**	(Qiao et al. 2017)
MgO/ KNO_3_	**450**	**300**	**0.2**	**1**	(Qiao et al. 2017)
MgO /Li_2_CO_3_	**450**	**325**	**1.08**	**1**	(Vu et al. 2014)
MgO-ZrO_2_	**700**	**30**	**1.15**	**1**	(Liu et al. 2008)
AC/MgO	**500**	**180**	**0.74**	**1**	(Liu and Green 2013)
AC-MgO	**325**	**25**	**1.12**	**-**	(Shahkarami et al. 2016)
MgO-based carbon adsorbents	**350**	**40**	**1.22**	**1**	(Heo and Park 2017)
20MgO/MCN	**500**	**25**	**1.15**	**1**	**Present work**
MgO	**550**	**25**	**0.74**	**1**	**Present Work**

## Conclusion

This work sheds light on the synthesis and utilization of a novel and recyclable MgO-based mesoporous carbon nitride adsorbent for efficient CO_2_ capture at ambient pressure and intermediate temperatures. MCN was utilized as a support material for developing a series of xMgO/MCN adsorbents with different MgO loadings (5–25 wt%). The as-obtained hybrids were appraised concerning their efficiency in the capture of CO_2_ from 10 vol% CO_2_ mixture gas with N_2_ using a fixed bed adsorber at atmospheric pressure. The CO_2_ capture amount firstly improved with increasing the MgO loading level until peaking to 1.15 mmol g^−1^ at 20% MgO due to the enhancement of the basic properties of the porous support material*.* Further increase in the amount of MgO to 25% adversely impacted the CO_2_ capture efficiency, presumably due to the blockage of the pores of MCN with excessive MgO*.* Besides, the capture process was found to be endothermic and the highest CO_2_ capture amount was obtained at 25 ºC nevertheless, a satisfactory CO_2_ adsorption capacity was achieved at 150 °C, demonstrating the effectiveness of composite for working over a wide operating temperature range. Notably, the 20MgO/MCN adsorbent revealed a fully reversible and stable CO_2_ capture performance over five runs. Thus, 20Mg/MCN revealed a good CO_2_ capture amount, wide operating temperature range, and superb reusable performance, rendering it attractive for practical CO_2_ capture applications.

## Data Availability

All data generated or analyzed during this study are included in this published article.
